# A Manual of Chemical Analysis as Applied to the Examination of Medicinal Chemicals

**Published:** 1883-04

**Authors:** 


**A Manual of Chemical Analysis as Applied to the Examination of Medicinal Chemicals.** A guide for the determination of their identity and quality, and for the detection of impurities and adulteration. For the use of pharmacists, physicians, druggists, manufacturing chemists and pharmaceutical and medical students. Third edition, thoroughly revised and greatly enlarged. By FREDERICK HOFFMAN, A. M., Ph. D., Public Analyst to the State of New York, and FREDERICK B. POWER, Ph. D., Professor of Chemistry in the Philadelphia College of Pharmacy. Philadelphia: Henry C. Lea's Son & Co. 1883.

The object of this excellent work is specifically stated in its title, and will be at once recognized as a valuable addition to our medical literature. At a time when the preparation of many of the medicinal agents is monopolized by parties who make a commercial commodity of valuable remedies, and of whose responsibility there is some reason to doubt, it is important that our druggists and pharmaceutists should have facilities at hand to assure themselves of the reliability of the agents they dispense. This work aims to be such a guide, and its publication will be hailed with pleasure by every druggist and practitioner who may desire *purity* in drugs, rather than *cheapness.* Part first of the work is devoted to an explanation of the operations involved in the application of simple tests and chemical examinations, designed to familiarize the student and inexperienced operator with the more important of the simple operations of the pharmaceutical laboratory, also a list of reagents and test solutions and a very concise course in qualitative and volumetric analysis, together with the general characters and the methods for the separation and recognition of some of the principal alkaloids and their allied principles. Part second includes medicinal chemicals and their preparation, their physical and and chemical characteristics, with directions for the examination of their quality and purity, and for their quantitative estimation. From this brief outline it will be seen that the publication of the work supplies a want felt by the profession to be necessary and claims a hearty endorsement. Measures that will facilitate the analysis of drugs, with a view to secure greater purity and reliability, are needed. The medical profession deeply feel this need. There is an increasing skepticism in regard to the preparations thrown upon the market by manufacturers, and urged by agents upon the notice of physicians. We commend, therefore, a work which affords our pharmaceutists and druggists the needed information and also a guide for the analysis of the agents they daily dispense.

